# Predicting the Onset of Anxiety Syndromes at 12 Months in Primary Care Attendees. The PredictA-Spain Study

**DOI:** 10.1371/journal.pone.0106370

**Published:** 2014-09-03

**Authors:** Patricia Moreno-Peral, Juan de Dios Luna, Louise Marston, Michael King, Irwin Nazareth, Emma Motrico, María Josefa GildeGómez-Barragán, Francisco Torres-González, Carmen Montón-Franco, Marta Sánchez-Celaya, Miguel Ángel Díaz-Barreiros, Catalina Vicens, Carlos Muñoz-Bravo, Juan Ángel Bellón

**Affiliations:** 1 Unidad de Investigación del Distrito Sanitario Málaga, Instituto de Investigación Biomédica de Málaga (IBIMA), Málaga, Spain; 2 Departamento de Bioestadística, Universidad de Granada, Granada, Spain; 3 Department of Primary care and Population Health, University College London, London, United Kingdom; 4 Mental Health Sciences, Faculty of Brain Sciences, University College London, London, United Kingdom; 5 Department of Primary care and Population Health, University College London, London, United Kingdom; 6 Universidad Loyola Andalucía, Sevilla, Spain; 7 Unidad Docente de Medicina Familiar y Comunitaria de La Rioja, Servicio Riojano de la Salud, Logroño, La Rioja, Spain; 8 Centro de Investigación Biomédica en Red de Salud Mental, Universidad de Granada, Granada, Spain; 9 Centro de Salud Casablanca, Instituto Aragonés de Ciencias de la Salud, Zaragoza, Spain. Departamento de Medicina y Psiquiatría, Universidad de Zaragoza, Zaragoza, Spain; 10 Directora Continuidad Asistencial Hospital Universitario Infanta Sofía, Madrid, Spain; 11 Centro de Salud Vecindario, Gerencia de Atención Primaria de Gran Canaria, Servicio Canario de Salud, Las Palmas, Spain; 12 Centro de Salud son Serra-La Vileta, Unidad Docente de Medicina Familiar y Comunitaria de Mallorca, Instituto Balear de la Salud, Palma de Mallorca, Illes Balears, Spain; 13 Departamento de Medicina Preventiva y Salud Pública, Universidad de Málaga, Málaga, Spain; 14 Centro de Salud El Palo, Servicio Andaluz de Salud, Málaga, Spain; Supportive care, Early DIagnosis and Advanced disease (SEDA) research group, United Kingdom

## Abstract

**Background:**

There are no risk algorithms for the onset of anxiety syndromes at 12 months in primary care. We aimed to develop and validate internally a risk algorithm to predict the onset of anxiety syndromes at 12 months.

**Methods:**

A prospective cohort study with evaluations at baseline, 6 and 12 months. We measured 39 known risk factors and used multilevel logistic regression and inverse probability weighting to build the risk algorithm. Our main outcome was generalized anxiety, panic and other non-specific anxiety syndromes as measured by the Primary Care Evaluation of Mental Disorders, Patient Health Questionnaire (PRIME-MD-PHQ). We recruited 3,564 adult primary care attendees without anxiety syndromes from 174 family physicians and 32 health centers in 6 Spanish provinces.

**Results:**

The cumulative 12-month incidence of anxiety syndromes was 12.2%. The predictA-Spain risk algorithm included the following predictors of anxiety syndromes: province; sex (female); younger age; taking medicines for anxiety, depression or stress; worse physical and mental quality of life (SF-12); dissatisfaction with paid and unpaid work; perception of financial strain; and the interactions sex*age, sex*perception of financial strain, and age*dissatisfaction with paid work. The *C-index* was 0.80 (95% confidence interval = 0.78–0.83) and the *Hedges'* g = 1.17 (95% confidence interval = 1.04–1.29). The *Copas shrinkage factor* was 0.98 and *calibration plots* showed an accurate goodness of fit.

**Conclusions:**

The predictA-Spain risk algorithm is valid to predict anxiety syndromes at 12 months. Although external validation is required, the predictA-Spain is available for use as a predictive tool in the prevention of anxiety syndromes in primary care.

## Introduction

Anxiety disorders occur in 6–12% of the general population [1]–[2], reaching 18.5% in primary care attendees [3]. Of patients with an anxiety disorder, 50% have another comorbid psychiatric condition such as depression or another anxiety disorder [4]. This results in a loss of 805 quality-adjusted life-years annually per 100,000 primary care patients, thus surpassing chronic physical illnesses such as cardiovascular diseases, hypertension, diabetes or chronic obstructive pulmonary disease [5].

Whilst great progress has been made in the development of effective psychological and pharmacological therapies for anxiety [6], these are either not accessed by most persons [7] or they are not applied correctly [8]. Accordingly, in addition to improving the process of the clinical care of patients with anxiety, recommendations suggest developing preventive programs. The latter require a clear understanding of the associated risk factors [9]. A prospective study of these risk factors, followed over time and modeled on large population-based samples, enables the development of a risk model for developing an anxiety disorder. This can inform individualized preventive therapies based on this perception of overall risk [10]. A risk algorithm (PredictA-Europe) to predict the onset of generalized anxiety and panic syndromes at 6 and 24 months already exists for European primary care attendees [11]. However, a prediction period of 6 months may be too short if we consider that the symptoms must last at least 6 months to fulfill the DSM-IV diagnosis of generalized anxiety disorder. Furthermore, a period greater than 12 months may include people who have developed more than one episode of anxiety. We therefore aimed to develop and internally validate a risk algorithm to predict the onset of anxiety syndromes at 12 months in primary care attendees.

## Method

### Design and setting

We undertook a prospective cohort study with evaluations at baseline, 6 and 12 months. Although this cohort was originally recruited with the aim of developing a risk model for the onset of major depression [12], in this analysis we aimed to predict the onset of anxiety syndromes.

The method has been described in detail elsewhere [12]. The predictA-Spain study was conducted with the participation of 32 health centers and 174 family physicians (FPs) distributed throughout Spain: Granada in southern Spain; Saragossa and La Rioja in northern Spain; Madrid, capital of Spain, situated in the center; Las Palmas in the Canary Islands; and Majorca in the Balearic Islands. Each health center covers a population of 15,000–30,000 inhabitants from a geographically defined area. The physicians in each health center work as a group, with extensive primary care teams. The Spanish National Health Service provides free medical cover to 95% of the population. Patients can visit their FP as often as they wish without having to pay for it, even when they do so for preventive reasons. Each patient is assigned to only one FP, who has gatekeeper functions. The health centers taking part cover urban and rural settings in each province.

### Sampling and exclusion criteria

In the six Spanish provinces, systematic random samples from physician appointment lists were taken at regular intervals of between four and six attendees with random starting points for each day. The FPs introduced the study to the selected patients and requested their permission before contacting the researcher. Participants who gave informed consent undertook a research interview within two weeks. The study population was recruited between October 2005 and February 2006. Exclusion criteria were an inability to understand or speak Spanish, severe mental disorder (e.g. psychosis, bipolar), dementia or severe neurological/sensory illness, terminal illness, the person was scheduled to be out of the city for more than three months during the 12 months of follow-up, and persons (representatives) who attended the surgery on behalf of the person who had the appointment.

### Variables

#### Outcome measure

The outcome of interest was anxiety syndromes over the preceding 6 months as defined by the anxiety section of the Primary Care Evaluation of Mental Disorders, Patient Health Questionnaire (PRIME-MD-PHQ) [13], [14]. The Spanish version of the PRIME-MD classifies patients who test positive for panic, generalized anxiety and other anxiety disorders [14]. The first two diagnoses match the DSM-IV criteria exactly, but the third is nonspecific. We used a dichotomous anxiety variable to indicate when any of the three diagnoses of anxiety were present in a given patient.

#### Potential risk factors measure

We selected 39 potential risk factors for which there was evidence of reliability and validity in the questionnaires used to evaluate them [12]. Baseline measurements were made of all the potential risk factors:

- Socio-demographic factors: (1) age, (2) sex, (3) marital status, (4) occupation, (5) employment status, (6) ethnicity, (7) nationality, (8) country of birth, (9) educational level, (10) income, (11) owner-occupier of their accommodation, (12) living alone or with others.
- Controls, demands and rewards for (13) paid and (14) unpaid work, using an adapted version of the job content instrument with 7 items each [12], [15].
- (15) Debt and financial strain by means of three questions with Likert responses [16]: 1) General financial strain: “How well would you say you are managing financially these days?” (4-Likert); 2) Basic financial strain: “How often does it happen that you do not have enough money to afford the kind of food or clothing you/your family should have?” (5-Likert); and 3) Coping with debts: “How much difficulty do you have in meeting the payments of household and other bills?” (6-Likert).
- (16) Physical and (17) mental well-being, assessed by the 12-item Short Form (SF-12) [17], [18] and (18) a question on the presence of long-standing illness, disability or infirmity.
- (19) Alcohol misuse, assessed by the Alcohol Use Disorders Identification Test (AUDIT) [19]–[21].
- (20) A screen for lifetime depression based on the first two questions of the Composite International Diagnostic Interview (CIDI) [22].
- (21) Lifetime use of recreational drugs (CIDI) [23].
- Brief questions on the quality of (22) sexual and (23) emotional relationships with a partner, adapted from a standardized questionnaire [24].
- (24) DSM-IV diagnosis of major depression in the preceding 6 months using the CIDI [23], [25], [26].
- (25) A question on taking medication for anxiety, depression or stress.
- Childhood experiences of (26) physical, (27) emotional or (28) sexual abuse [27].
- (29) Nature and strength of spiritual beliefs [28].
- (30) Presence of serious physical or psychological disorder, or substance misuse problems, or any serious disability in persons who were close friends or relations of participants.
- (31) Difficulty getting on with people and maintaining close relationships, assessed using questions from a social functioning scale [29].
- (32) History of serious psychological problems or (33) suicide in first-degree relatives [30].
- (34) Satisfaction with the neighborhood and (35) perceived safety inside/outside the home using questions from the Health Survey for England [31].
- (36) Threatening events in the preceding 6 months using the List of Threatening Experiences Questionnaire [32], [33].
- (37) Experiences of discrimination in the preceding 6 months on grounds of sex, age, ethnicity, appearance, disability, or sexual orientation, using questions from a European study [34].
- (38) Adequacy of social support from family and friends [35].
- (39) Two questions about smoking habits [36].

### Statistical Analysis

Participants with missing anxiety diagnoses at both follow-up points (at 6 and 12 months) were excluded. We also excluded those with missing anxiety diagnoses at one follow-up point who had no anxiety syndromes at the other. However, we included patients with anxiety syndromes at one follow-up point and missing diagnoses at the other (at 6 or 12 months), as they met the outcome criterion of anxiety at some point over the 12 months. We performed multilevel logistic regression with cumulative anxiety incidence as the dependent variable and health center as a random component. To test the hierarchical data structure we used the likelihood-ratio test of the null model taking cumulative incidence of anxiety syndromes at 12 months as the dependent variable and health center as a random factor versus usual logistic regression [Chi2 = 28.94; p<0.0001]. The Intraclass Correlation Coefficient for Health Center was 0.082 (95% Confidence Interval: 0.039–0.166). The likelihood-ratio test of the null model with the variable doctor as a random factor versus usual logistic regression was also significant [Chi2 = 12.81; p = 0.0002]. The Intraclass Correlation Coefficient of the variable doctor was 0.091 (95% Confidence Interval: 0.044–0.180). We then checked the likelihood-ratio test of the null model with health center and doctor as random factors versus the null model with only health center [Chi2 = 0.71; p = 0.2002]. We therefore decided to use multilevel logistic regression with health center as the random component.

We selected variables using a threshold for inclusion of p<0.20 to ensure that information lost as a result of exclusion of a variable from the equation was minimal [37]. From the model thus obtained, those variables with p>0.05 were extracted step by step to obtain a more parsimonious model. The usefulness of including first-degree interactions was considered, especially the interaction age*sex because it has been found previously in depression [38], [39] and anxiety [40], as well as the different combinations of age and sex with the other variables included in the model. We decided to include an interaction in the model when the likelihood ratio test was significant at p<0.05. We used inverse probability weighting [41], [42] to adjust for a possible attrition bias due to participants lost to follow-up. All reported P values were two-sided.

The ability to distinguish those who would develop anxiety syndromes from those who would not was assessed using the *C-index* [43]. To compare the discriminative validity between two risk algorithms we performed the test for two correlated C-*index*. Prediction models derived with multivariable regression analysis are known for overestimating regression coefficients. We used a calculation proposed by *Copas* [44] to estimate overfitting of our prediction models. We calculated effect sizes using *Hedges' g* [45]. Calibration, which is the agreement between the observed proportions of major depressive disorder and the predicted risks, was studied with calibration plots taking deciles of risk. We conducted all analyses using STATA, release 12 [46].

### Ethics Statement

The predictA-Spain study has been conducted according to the principles expressed in the Declaration of Helsinki. This study complies with the Code of Ethics of the World Medical Association and was approved by the ethics committees: Ethics Committee on Human Research of the University of Granada, Ethics and Research Committee of Primary Health District of Malaga, Ethics Committee for Clinical Research of Aragon (CEICA). Research assistants explained to patients the predictA-Spain study in detail, their commitments and rights, and answered all questions that patients wanted to ask. All the participants read an information sheet and signed consent forms to take part in the study.

## Results

**Figure 1:**
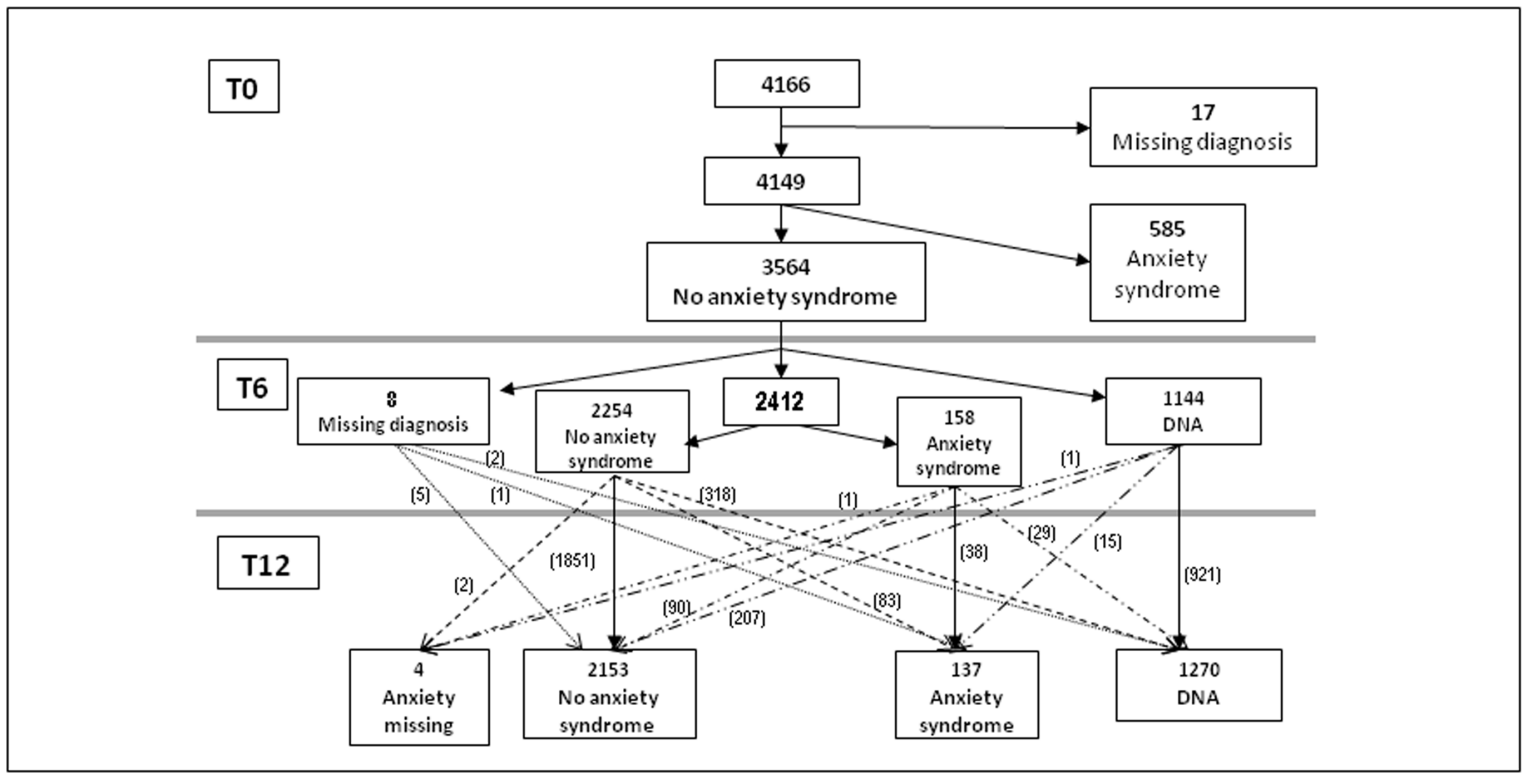
Flow chart of patients through the predictA-Spain study and numbers becoming anxious. Footnote to Figure 1: DNA: did not attend; T0, T6 and T12: baseline, 6 and 12 months interview. Anxiety syndromes measured by the Primary Care Evaluation of Mental Disorders, Patient Health Questionnaire (PRIME-MD-PHQ). ^12,13^

Of the 6,299 primary care attendees approached, 1,251 (19.9%) were excluded: 506 (8.03%) were outside the age range (18–75 years); 446 (7.1%) were either representatives of patients or did not attend the appointment; 156 (2.5%) had severe mental disorder, dementia or severe neurological/sensory illness; 63 (1.0%) terminal illness; 47 (0.75%) trouble communicating in Spanish; and 33 (0.52%) were scheduled to be out of the city for longer than three months during the 12 months of follow-up. Of the remaining 5,048 patients asked to take part in the study 4,166 (82.5%) gave their consent. These were then interviewed at baseline, but 585 (14.0%) had a positive diagnosis of anxiety syndrome (by PRIME-MD-PHQ) and 17 (0.41%) had a missing diagnosis, so they were also excluded. Thus, our at-risk population comprised 3,564 patients (Figure 1).

Patients' socio-demographic characteristics are shown in Table 1.

**Table 1:** Demographic characteristics of participants without anxiety at baseline (population at risk).

Demographic characteristics	Granada	Saragossa	Madrid	La Rioja	Majorca	Las Palmas	Total
**Participants without anxiety**, n(%)	662 (18.6)	688 (19.3)	642 (18)	706 (19.8)	575 (16.1)	291 (8.2)	3564 (100)
**Age** (**years**), mean (standard deviation)	49.83 (16.47)	46.48 (15.5)	50.34 (16)	49.21 (15.7)	50.1 (15.8)	43.11 (14.47)	48.65 (15.91)
**Sex**, n(%)							
Female	465 (70.24)	421 (61.19)	420 (65.42)	444 (62.89)	355 (61.74)	199 (68.38)	2304 (64.6)
Male	197 (29.76)	267 (38.81)	222 (34.58)	262 (37.11)	220 (38.26)	92 (31.62)	1260 (35.4)
**Marital status**, n(%)							
Married	449 (67.82)	443 (64.39)	429 (66.82)	466 (66.01)	365 (63.48)	145 (49.83)	2297 (64.5)
Separated	21 (3.17)	22 (3.2)	27 (4.21)	28 (3.97)	35 (6.09)	25 (8.59)	158 (4.4)
Divorced	5 (0.76)	13 (1.89)	14 (2.18)	9 (1.27)	18 (3.13)	14 (4.81)	73 (2)
Single	127 (19.18)	178 (25.87)	134 (20.87)	159 (22.52)	113 (19.65)	91 (31.27)	802 (22.5)
Widowed	60 (9.06)	32 (4.65)	38 (5.92)	44 (6.23)	44 (7.65)	15 (5.15)	233 (6.5)
Missing	0	0	0	0	0	1 (0.34)	1 (0.1)
**Household status**, n(%)							
Not living alone	601 (90.79)	639 (92.88)	589 (91.74)	641 (90.79)	507 (88.17)	275 (94.5)	3252 (91.2)
Living alone	61 (9.21)	49 (7.12)	53 (8.26)	65 (9.21)	68 (11.83)	16 (5.5)	312 (8.8)
**Education**, n(%)							
Higher education	84 (12.69)	106 (15.41)	61 (9.5)	113 (16.01)	33 (5.74)	41 (14.09)	438 (12.3)
Secondary	117 (17.67)	180 (26.16)	156 (24.3)	150 (21.25)	98 (17.04)	75 (25.77)	776 (21.8)
Primary	243 (36.71)	324 (47.09)	254 (39.56)	392 (55.52)	344 (59.83)	127 (43.64)	1684 (47.3)
Trade/other	218 (32.93)	78 (11.34)	171 (26.64)	50 (7.08)	100 (17.39)	48 (16.49)	665 (18.7)
Missing	0	0	0	1 (0.14)	0	0	1 (0.1)
**Employment**, n(%)							
Employed	229 (34.59)	364 (52.91)	296 (46.11)	349 (49.43)	221 (38.43)	164 (56.36)	1623 (45.5)
Unemployed	50 (7.55)	40 (5.81)	26 (4.05)	46 (6.52)	36 (6.26)	35 (12.03)	233 (6.5)
Retired	141 (21.3)	111 (16.13)	141 (21.96)	154 (21.81)	118 (20.52)	23 (7.9)	688 (19.3)
Unable to work	51 (7.7)	15 (2.18)	35 (5.45)	7 (0.99)	99 (17.22)	14 (4.81)	221 (6.2)
Looking after family	161 (24.32)	130 (18.9)	133 (20.72)	132 (18.7)	95 (16.52)	44 (15.12)	695 (19.5)
Full-time student	27 (4.08)	26 (3.78)	8 (1.25)	17 (2.41)	5 (0.87)	7 (2.41)	90 (2.5)
Other	1 (0.15)	1 (0.15)	1 (0.16)	1 (0.14)	1 (0.17)	4 (1.37)	9 (0.3)
Missing	2 (0.3)	1 (0.15)	2 (0.31)	0	0	0	5 (0.1)
**Country of birth**, n(%)							
Spain	644 (97.28)	654 (95.06)	596 (92.83)	668 (94.62)	531 (92.35)	254 (87.29)	3347 (93.9)
Other	17 (2.57)	33 (4.8)	38 (5.92)	38 (5.38)	38 (6.61)	31 (10.65)	195 (5.5)
Missing	1 (0.15)	1 (0.15)	8 (1.25)	0	6 (1.04)	6 (2.06)	22 (0.6)
**Ethnicity**, n(%)							
White European	646 (97.58)	567 (82.41)	618 (96.26)	668 (94.62)	557 (96.87)	288 (98.97)	3344 (93.8)
Other ethnicity	11 (1.66)	7 (1.02)	22 (3.43)	22 (3.12)	13 (2.26)	3 (1.03)	78 (2.2)
Missing	5 (0.76)	114 (16.57)	2 (0.32)	16 (2.27)	5 (0.87)	0	142 (4)
**Income**, n(%)							
<15000€	386 (58.31)	266 (38.66)	335 (52.18)	212 (30.03)	258 (44.87)	111 (38.14)	543 (15.2)
15000€–30000€	171 (25.83)	237 (34.45)	223 (34.74)	174 (24.65)	175 (30.43)	68 (23.37)	1568 (44)
>30000€	51 (7.7)	104 (15.12)	59 (9.19)	97 (13.74)	73 (12.7)	21 (7.22)	1048 (29.4)
No response	54 (8.16)	81 (11.77)	25 (3.89)	223 (31.59)	69 (12)	91 (31.27)	405 (11.4)

Of the 3,564 patients, 2,420 (68%) were interviewed at 6 months and 2,294 (64.4%) at 12 months. The variables associated with drop-outs were province (Madrid and Majorca), sex (male), lower age, lower educational level, seldom having enough money to afford food or clothing (basic financial strain), discrimination due to age, daily smoker, and lifetime depression (see Table 2). There were only nine missing values for predictor variables in the final models to predict the onset of anxiety syndromes. The cumulative 12-month incidence of anxiety syndromes was 12.2% (panic disorder 4.5%, generalized anxiety disorder 4.1%, other anxiety disorders 5%).

**Table 2:** Model to predict drop-out.

	*Odds Ratio	95% confidence interval	p
**Province**			
Granada			
Saragossa	1.302	0.840–2.018	0.238
Madrid	1.868	1.191–2.930	0.007
Logroño	1.526	0.988–2.357	0.057
Majorca	3.522	2.240–5.537	<0.001
Las Palmas	1.711	0.996–2.936	0.052
**Sex**			
Female			
Male	1.382	1.189–1.607	<0.001
**Age** (years)	0.986	0.981–.992	<0.001
**Education**			
Beyond secondary education			
Secondary education	1.240	0.960–1.600	0.099
Primary education	1.328	1.043–1.692	0.022
Incomplete primary education or illiterate	1.594	1.178–2.158	0.002
**Enough money to afford food or clothing**			
Always			
Often	1.122	0.912–1.381	0.275
Sometimes	0.982	0.768–1.256	0.885
Seldom	2.141	1.015–4.517	0.046
Never	0.650	0.286–1.479	0.304
**Discrimination due to age**			
No			
Yes	1.890	1.169–3.054	0.009
**Daily smoker**			
Non or ex-smoker			
Up to 10 cigarettes	1.199	0.941–1.528	0.142
From 11 to 20 cigarettes	1.316	1.034–1.676	0.026
From 21 to 30 cigarettes	0.873	0.573–1.331	0.529
More than 30 cigarettes	1.391	0.773–2.502	0.271
**Lifetime depression**			
No			
Yes	1.22	1.048–1.415	0.010

(*) Multi-level logistic regression with health center as a random component. We selected variables included in the final model from the 39 measured in this study using a threshold for inclusion of p<0.20 in bivariate regression. From the model thus obtained, those variables with p>0.05 were extracted step by step to obtain a more parsimonious model.

**Figure 2:**
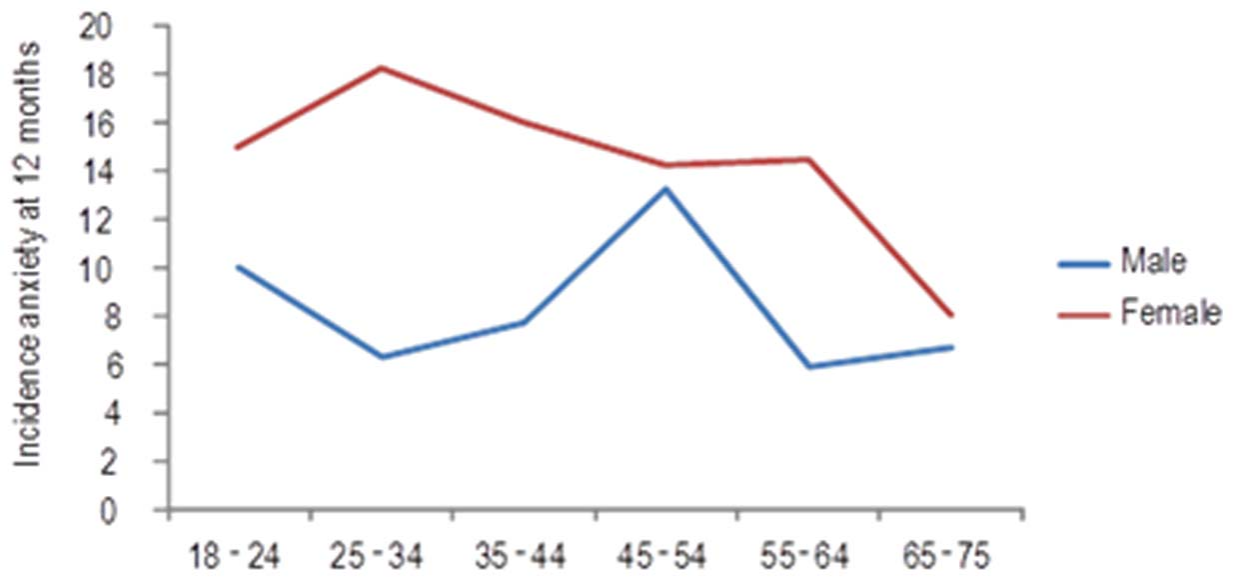
Incidence of anxiety by age and sex.

**Figure 3:**
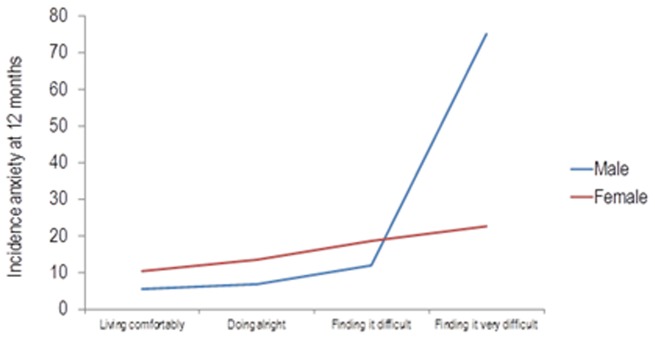
Incidence of anxiety by sex and level of financial strain.

**Figure 4:**
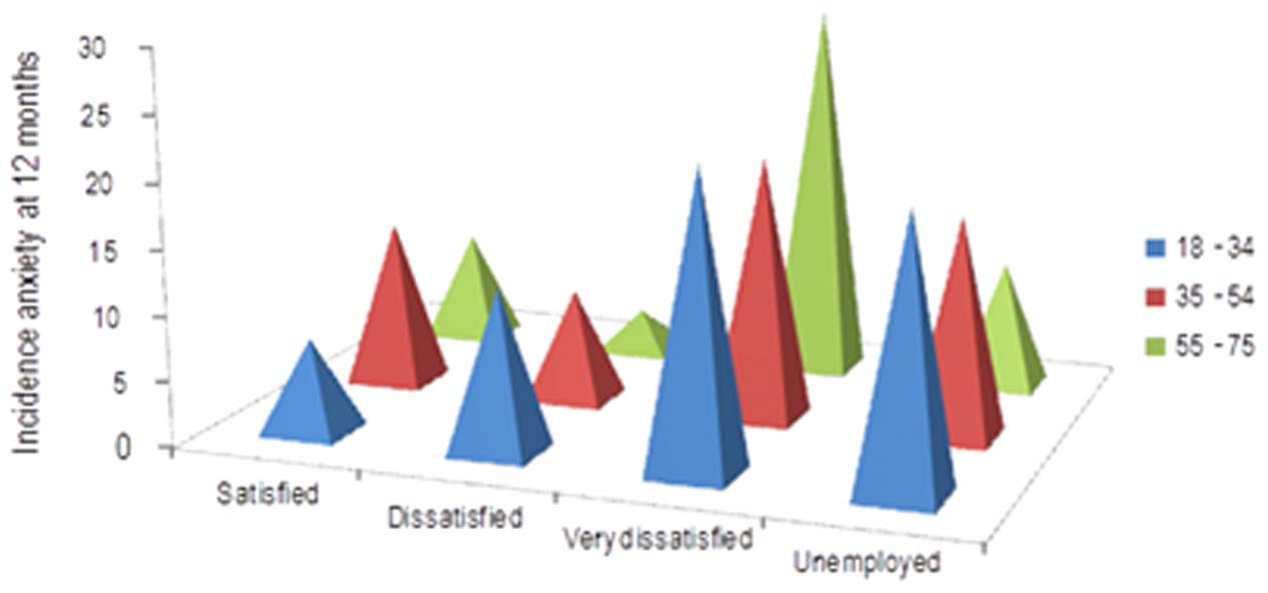
Incidence of anxiety by age and dissatisfaction at work.

The final model to predict anxiety syndromes included 9 variables and 3 interactions (Table 3): province; sex (female); lower age; taking medicines for anxiety, depression or stress; worse physical and mental life quality (SF-12); dissatisfaction with paid and unpaid work; perceived general financial strain; and the interactions sex*age, sex*perceived financial strain, and age*dissatisfaction with paid work. From the 15 interactions tested we selected the three that had a p<0.05. Firstly, we compared the nine-variable model plus the interaction sex*age and the nine-variable model plus the interactions sex*age and sex*financial strain [*chi2* = 13.90; *p* = 0.003]. Secondly, we compared the nine-variable model plus the interaction sex*age with the nine-variable model plus the interactions sex*age and age*paid work [*chi2* = 11.55; *p* = 0.009]. Thirdly, we compared the nine-variable model plus the interactions sex*age and sex*financial strain with the nine-variable model plus the interactions sex*age, sex*financial problems, and age*paid work [*chi2* = 11.76; *p* = 0.008]. The likelihood ratios for the interactions analyzed are described in Table 4 and charts of the three interactions are shown in Figures 2–3–4.

**Table 3:** Models^a^ for predicting the onset of anxiety at 12 months (N = 2.103).

	Model without interactions^b^			Model with interactions^c^			
Risk factors	ß	S.E.	p	ß	ß^d^	S.E.	p
**Constant Province**	1.647	0.769	0.032	0.475	0.238	1.578	0.161
Granada							
Saragossa	0.984	0.233	<0.001	1.064	1.042	0.241	<0.001
Madrid	−0.976	0.246	<0.001	−0.941	−0.921	0.275	0.001
Logroño	−0.343	0.260	0.187	−0.333	−0.326	0.287	0.246
Majorca	0.699	0.230	0.002	0.741	0.726	0.246	0.003
Las Palmas	−0.356	0.334	0.287	−0.333	−0.326	0.345	0.333
**Sex**							
Female							
Male	−0.453	0.169	0.008	−1.735	−1.699	0.962	0.071
**Age** (years)	−0.023	0.006	<0.001	0.010	−0.010	0.013	0.459
**Financial strain**							
Living comfortably							
Doing alright	0.484	0.247	0.050	0.498	0.488	0.291	0.087
Finding it difficult	0.629	0.310	0.042	0.545	0.534	0.374	0.144
Finding it very difficult	0.943	0.409	0.021	0.299	0.293	0.525	0.569
**Taking psychotropic drugs**							
No							
Yes	0.614	0.244	0.012	0.609	0.596	0.241	0.011
**Physical health** (SF-12, range = 0–100)							
Each point on subscale score	−.032	0.008	<0.001	−0.033	−0.032	0.008	<0.001
**Mental health** (SF-12, range = 0–100)							
Each point on subscale score	−.046	0.009	<0.001	−0.049	−0.048	0.009	<0.001
**Dissatisfaction with paid work**							
Satisfied							
Dissatisfied	−0.017	0.238	0.943	2.245	2.198	1.041	0.031
Very dissatisfied	0.879	0.255	0.001	2.463	2.412	0.928	0.008
Jobless	0.429	0.246	0.081	2.198	2.152	0.527	<0.001
**Dissatisfaction with unpaid work**							
Satisfied							
Dissatisfied	0.654	0.220	0.767	0.064	0.063	0.229	0.779
Very dissatisfied	1.042	0.375	0.005	1.129	1.105	0.345	0.001
No response	0.188	0.279	0.500	0.144	0.141	0.274	0.598
Sex*age				0.027	0.026	0.014	0.059
Sex*financial strain							
Living comfortably							
Doing alright				−0.157	−0.154	0.784	0.841
Finding it difficult				0.133	0.130	0.980	0.892
Finding it very difficult				4.049	3.965	2.231	0.070
Age*dissatisfaction with paid work							
Satisfied							
Dissatisfied				−0.056	−0.055	0.026	0.035
Very dissatisfied				−0.037	−0.036	0.023	0.102
Jobless				−0.043	−0.042	0.013	0.001

a Model derived weighting for the inverse probability of remaining in the follow-up to 12 months.

b Discriminative validity: C-Index: 0.7917 (C.I.95% = 0.7653–0.8181) and Effect size (Hedges' g): 1.1058 (C.I.95% = 0.9854–1.2261). Overfitting estimate: Copas' shrinkage factor: 0.9162.

c Discriminative validity: C-Index: 0.80 (C.I.95% = 0.78–0.83) and Effect size (Hedges' g): 1.17 (C.I.95% = 1.04–1.29). Overfitting estimate: Copas' shrinkage factor: 0.9793.

d Coefficient after Copas shrinkage. S.E.: Standard error.

**Table 4:** Comparison of the base model with the models including each of the interactions tested.

Interactions	Likelihood-ratios between model without interactions and model with each interaction	
	*chi2*	*P*
Sex*province#		
Sex*age	7.12	**0.008**
Sex*financial strain	12.98	**0.005**
Sex*taking psychotropic drugs	2.60	0.107
Sex*physical health	0.00	0.945
Sex*mental health	1.31	0.253
Sex*paid work	2.70	0.441
Sex*unpaid work	1.42	0.702
Age*province	4.24	0.515
Age*financial strain	3.14	0.371
Age*taking psychotropic	0.02	0.889
Age*physical health	0.23	0.630
Age*mental health	2.12	0.145
Age*paid work	11.01	**0.012**
Age*unpaid work	1.36	0.716

# The model sex*province did not converge.

Concerning the interaction sex*age, whereas the tendency for the incidence of anxiety in women could be drawn with a more or less descending line with effect from the age of 25 years, the tendency for the men rose from the age of 25 years up to 54 years, with the peak between 45–54 years (Figure 2).

The interaction sex*financial strain showed that in men with many financial problems the risk for the incidence of anxiety was increased four-fold (Figure 3).

The interaction age*dissatisfaction at work had an antagonistic effect, with dissatisfaction at work in the younger patients increasing the incidence of anxiety (Figure 4).

**Figure 5:**
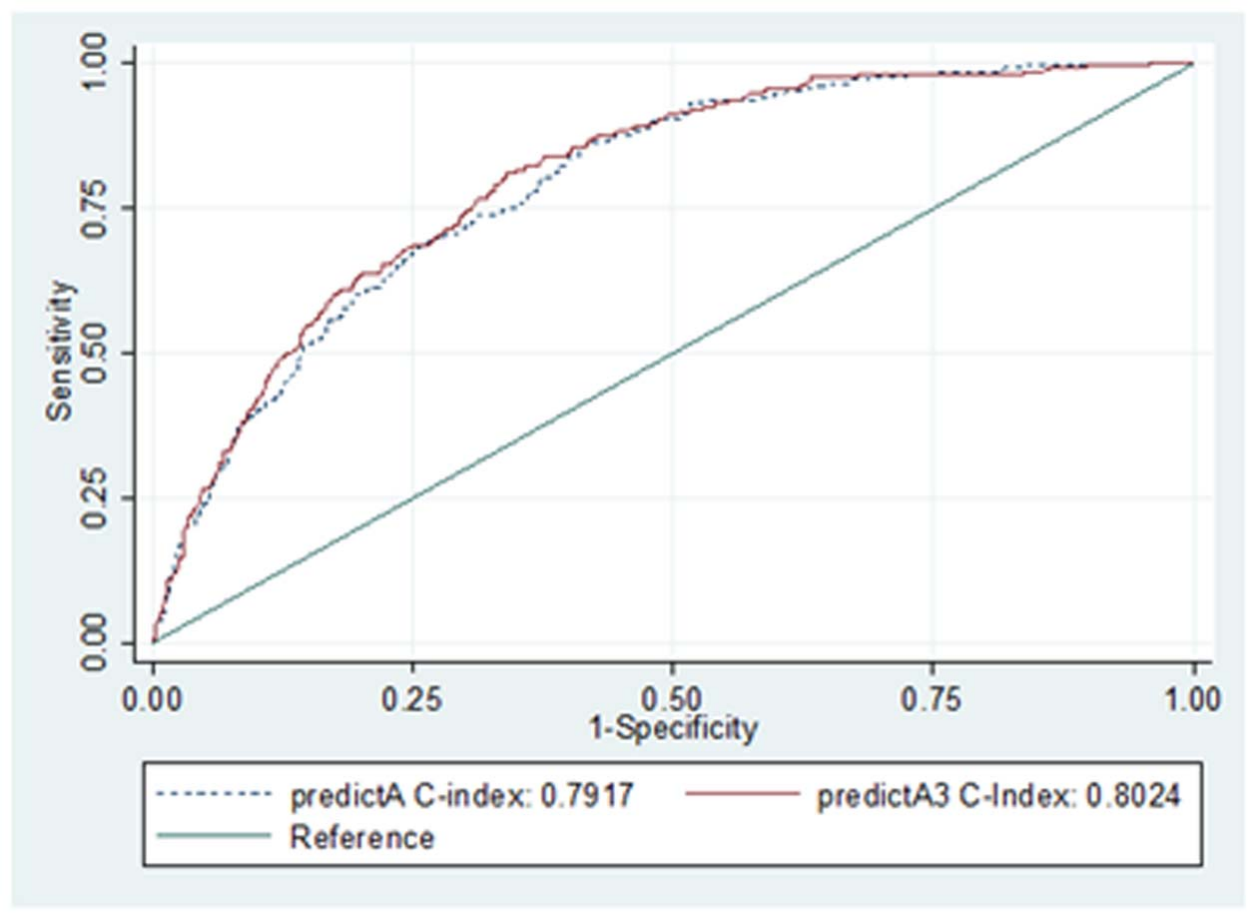
Area under the ROC curve for the models with/without interactions. Footnote toFigure 5: *predictA: model without interactions predictA3: model with 3 interactions*.

The *shrinkage factor* was 0.9793 (shrinkage = 1 indicates that there is no overestimation). The *C-index* and *effect size* (*Hedges' g*) were 0.80 [95% confidence interval (CI) = 0.78–0.83] and 1.17 (95%CI = 1.04–1.29), respectively, and the C-*index* data are represented in Figure 5. There was a slight improvement in the C-index in the model with interactions (0.8024; 95%CI = 0.7763–0.8284) versus the model without interactions (0.7917; 95%CI = 0.7653–0.8181); Chi^2^ = 5.11(DF = 1) and p = 0.0238 (Figure 5).

We incorporated depression (measured by the CIDI at baseline) as a risk factor candidate to be included in the model to predict the onset of anxiety syndromes but, although it was statistically significant in bivariate analysis (OR = 2.83; 95% CI = 1.70–4.71; p<0.0001), it did not remain in the final model after adjustment. If we removed from the final model the variables “worse mental life quality (SF-12-mental) and taking medication for anxiety, depression or stress” and then included major depression (CIDI), the latter reached statistical significance (OR = 1.88; 95%CI = 1.18–2.99; p = 0.008); but if we just removed only one of them (SF12-mental or taking psychotropic agents) then major depression was not significant. Our final model was clearly more discriminant (C-index = 0.80; 95%CI = 0.78–0.83) than the model excluding SF12-mental and taking psychotropic agents and including major depression (C-index = 0.77; 95%CI = 0.74–0.79); with the test for its difference being statistically significant [chi2(1) = 14.54; P = 0.0001].

**Figure 6:**
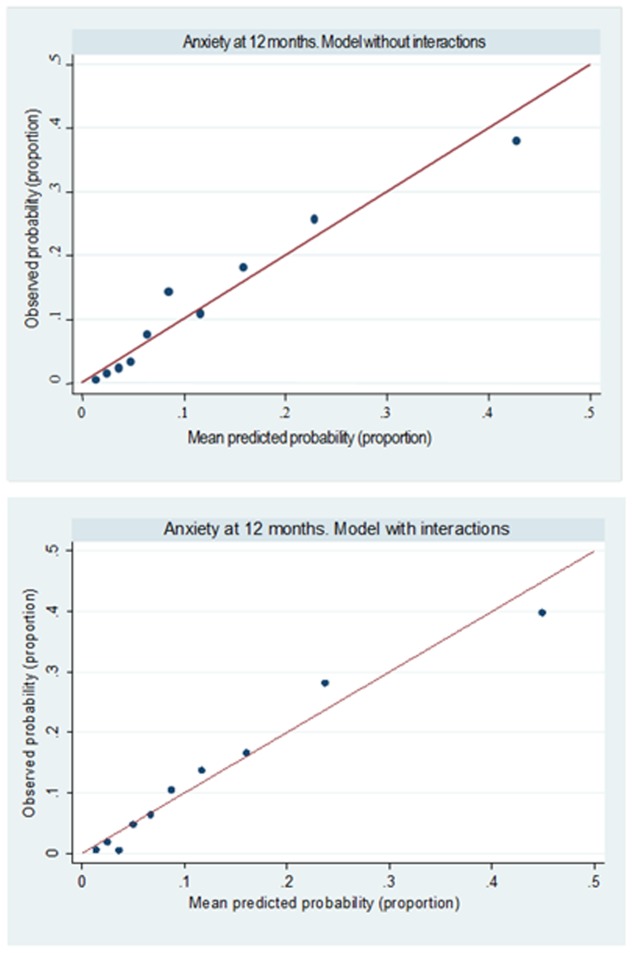
Calibration plots (mean predicted probability versus observed probability of anxiety within deciles of predicted risk) of the predictA-Spain risk algorithm.

The calibration showed an accurate goodness of fit (Figure 6). The predicted probability cut-point of 10% was associated with the greatest Youden's J statistic (J =  Sensitivity + Specificity -1), which had good sensitivity (80.2%) but poor specificity (66.5%). The predicted probability of 11% reached a specificity of 70% and a sensitivity of 75%; while the predicted probability of 13% had more specificity (75%) than sensitivity (approximately 69%), see Table 5.

**Table 5:** Predicted probability cut-points to predict anxiety syndromes at 12 months and their associated validity.

Predicted probability cut-points	Sensitivity (%)	Specificity (%)	LR+	LR-
≥0.0817	84.8	59.9	2.12	0.25
≥0.0928	82.5	64.1	2.30	0.27
≥0.0998*	80.2	66.5	2.39	0.30
≥0.1053	77.4	67.8	2.41	0.33
≥0.1108	74.7	69.8	2.48	0.36
≥0.1147	72.4	71.0	2.50	0.40
≥0.1204	70.0	72.9	2.60	0.41
≥0.1291	68.5	75.1	2.75	0.42
≥0.1442	65.4	80.0	2.96	0.44
≥0.1699	60.3	82.7	3.48	0.48
≥0.1860	55.3	84.7	3.62	0.53

LR+ Likelihood ratio of the positive test; LR- Likelihood ratio of the negative test.

*Cutpoint where Youden's J statistic (J  =  Sensitivity + Specificity -1) was greater.

Differences (variations in coefficients >10%) were found between the final model and the same model weighted for the inverse probability of remaining in the follow-up to 12 months (Table 6). Examples of the kinds of participants scoring at increasing levels of predicted probability of anxiety syndromes are shown in Table 7.

**Table 6:** Weighted and unweighted models* by the inverse probability of remaining in the follow-up to 12 months.

	Model weighted			Model unweighted		
Risk factors	ß	S.E.	p	ß	S.E.	P
**Constant Province**	0.475	1.578	0.161	0.265	0.893	0.767
Granada						
Saragossa	1.064	0.241	<0.001	0.970	0.242	<0.001
Madrid	−0.941	0.275	0.001	−1.010	0.309	0.001
Logroño	−0.333	0.287	0.246	−0.405	0.287	0.157
Majorca	0.741	0.246	0.003	0.672	0.263	0.011
Las Palmas	−0.333	0.345	0.333	−0.345	0.359	0.336
**Sex**						
Female						
Male	−1.735	0.962	0.071	−1.560	0.886	0.078
**Age** (years)	0.010	0.013	0.459	0.014	0.016	0.373
**Financial problems**						
Living comfortably						
Doing alright	0.498	0.291	0.087	0.454	0.340	0.182
Finding it difficult	0.545	0.374	0.144	0.507	0.376	0.178
Finding it very difficult	0.299	0.525	0.569	0.226	0.652	0.729
**Taking psychotropic drugs**						
No						
Yes	0.609	0.241	0.011	0.544	0.176	0.002
**Physical health** (SF-12)						
Each point on subscale score	−0.033	0.008	<0.001	−0.030	0.007	<0.001
**Mental health** (SF-12)						
Each point on subscale score	−0.049	0.009	<0.001	−0.048	0.007	<0.001
**Dissatisfaction with paid work**						
Satisfied						
Dissatisfied	2.245	1.041	0.031	2.334	0.953	0.014
Very dissatisfied	2.463	0.928	0.008	2.368	1.094	0.030
Jobless	2.198	0.527	<0.001	2.072	0.783	0.008
**Dissatisfaction with unpaid work**						
Satisfied						
Dissatisfied	0.064	0.229	0.779	0.086	0.188	0.646
Very dissatisfied	1.129	0.345	0.001	1.054	0.275	<0.001
Not response	0.144	0.274	0.598	0.186	0.284	0.511
Sex*age	0.027	0.014	0.059	0.025	0.012	0.039
Sex*financial problems						
Living comfortably						
Doing alright	−0.157	0.784	0.841	−0.276	0.724	0.703
Finding it difficult	0.133	0.98	0.892	0.043	0.793	0.956
Finding it very difficult	4.049	2.231	0.070	3.754	1.607	0.020
Age*paid work						
Satisfied						
Dissatisfied	−0.056	0.026	0.035	−0.059	0.023	0.009
Very dissatisfied	−0.037	0.023	0.102	−0.035	0.025	0.171
Unemployed	−0.043	0.013	0.001	−0.042	0.017	0.014

**S.E.**: Standard error. (*) Multi-level logistic regression with health center as a random component.

**Table 7:** Examples of predicted probabilities of anxiety at baseline.

Risk score	Risk profile
2.9%	A 61-year-old man living in Madrid
	Very dissatisfied with paid work and satisfied with unpaid work
	*SF-12 mental scale score 61.1
	SF-12 physical scale score 32.3
	Doing alright (financial problems)
	Does not take medication for anxiety, depression or stress
12.9%	A 22-year-old man living in Granada
	Jobless and dissatisfied with unpaid work
	SF-12 mental scale score 32.9
	SF-12 physical scale score 45.8
	Doing alright (financial problems)
	Does not take medication for anxiety, depression or stress
34.6%	A 40-year-old woman living in Las Palmas
	Jobless and satisfied with unpaid work
	SF-12 mental scale score 38.2
	SF-12 physical scale score 26.8
	Doing alright (financial problems)
	Taking medication for anxiety, depression or stress
84.1%	A 37-year-old woman living in Saragossa
	Very dissatisfied with paid work and dissatisfied with unpaid work
	SF-12 mental scale score 23.8
	SF-12 physical scale score 40.2
	Doing alright (financial problems)
	Taking medication for anxiety, depression or stress

(*)The possible range of the SF-12 is 0–100. High scores indicate good health/well-being. Mean (Standard Deviation) Short Form 12 (SF-12) mental and physical subscale scores for Spain are 47.1 (12.4) and 43.8 (11.4), respectively [39].

## Discussion

The predictA-Spain risk algorithm is valid. To our knowledge, it is the first algorithm that has been developed and internally validated to predict the onset of anxiety syndromes at 12 months in primary care attendees. From the *shrinkage factor* obtained, it can be deduced that coefficients were minimally over-estimated in the internal validation process. However, external validations are required to apply this risk algorithm in different populations. We used multi-level regression because of the hierarchical structure of the data. This approach improves the accuracy of estimates of coefficients and standard errors [47]. Our large sample size and the number of events (people developing anxiety syndromes) per variable included in the model reduced the risk of selecting unimportant variables and failing to include important variables [48]. However, the sample size was not large enough to address external validation in this study, derivating the algorithm in some provinces and validating in the others. Though our sample possibly under-represented patients who attend infrequently [49], frequent attendees are more likely to suffer from anxiety disorders [50] and therefore are most in need of prevention.

Important differences were seen between the predictA-Spain models with and without inverse probability weighting, indicating that loss to follow-up might lead to attrition bias and that this strategy could provide unbiased estimates of coefficients, even in the presence of attrition bias [41].

The questionnaire used to evaluate our outcome, PRIME-MD, has good reliability and validity indices [14], but we cannot rule out classification bias. Moreover, we only considered generalized anxiety and panic disorders as defined by the PRIME-MD. Our data do not concern other anxiety disorders such as post-traumatic stress, obsessive compulsive or phobic anxiety disorders.

In terms of C-index and effect size (Hedges' g), the predictA-Spain risk score compares favorably with the predictA-Europe, although the differences between the two risk scores might be explained in part by the greater homogeneity of the sample in Spain. The predictA-Spain is also similar to other risk algorithms for the onset of major depression [39], [51] as well as for risk indices for cardiovascular events [52].

The predictA-Spain risk algorithm shared some risk factors with the predictA-Europe [11] (sex, age, physical and mental quality of life, and dissatisfaction with paid and unpaid work) but differed in others: (1) taking medicines for anxiety, depression or stress, (2) perceived financial problems, and (3) the interactions. Moreover, the variable dissatisfaction with paid and unpaid work was measured differently (a dichotomous combined variable in the predictA-Europe and two separate scales in the predictA-Spain). The first difference (1) might, in theory, be because that variable was not included as a candidate in the stepwise selection process in predictA-Europe.

We are quite sure that our population at risk had no anxiety syndromes at baseline, at least during the preceding 6 months as defined by the anxiety section of the PRIME-MD. However, we cannot rule out that some patients had suffered anxiety syndromes prior to that date. In addition, our population at risk could have had depression or dysthymia at baseline or before. For any of these reasons, patients might be taking (appropriately or inappropriately) antidepressants and/or anxiolytics at baseline. There may also be patients (without diagnostic criteria for anxiety or depression disorders) who had insomnia and for whom a doctor could have prescribed pills to sleep. In the case of taking anxiolytics over a long time, along with insomnia, a percentage of the patients could also have an addiction disorder. Moreover, the question is phrased in such a way that it might include those taking anxiolytics-antidepressants or other medicines (vitamins, placebos, etc.), often inadequately, for minor emotional problems.

Including this variable in the final model does not mean that taking psychotropic drugs causes anxiety syndromes. If our objective is to obtain a risk algorithm, all variables potentially associated with the outcome, not necessarily causally, can be considered in order to predict as accurately as possible [53]. We believe that when patients respond that they are taking medication for anxiety, depression or stress, it should be understood as an intermediate variable related to one or more mental health disorder, such as depression, dysthymia, insomnia, addictions, personality disorders, adjustment disorders or other minor emotional problems. We have checked this with our data and this may be true at least in the case of major depression (see the results section); additionally, suffering other mental disorders is a well known risk factor for anxiety disorders [54].

One hypothesis to explain the inclusion of this variable in the predictA-Spain risk algorithm might be that Spanish patients have a tendency to ask their FPs for more psychotropic drugs for emotional problems encountered in everyday life and Spanish FPs tend to give them more medication. There is some support for this hypothesis because Spain is among those European countries having a higher use of psychotropic drugs [55].

The question “taking medication for anxiety, depression or stress” is a little ambiguous, but it is a good and independent predictor of the onset of anxiety syndromes and it is also very easy to obtain an answer.

The second difference (2) may be related with the fact that the economic indicators for Spain are below those of the European mean [56]. Finally, (3) the fact that the interactions were not included in the predictA-Europe might be explained because only the interactions between time and variables were explored in the model [11].

The selection of risk factors was performed with the aim of building a predictive model for major depression. However, it is known that anxiety and depression share most of their risk factors [11], [39], [51], [57]. The predictA-Spain shared seven risk factors with the predictD-Spain [39] risk algorithm to predict the onset of major depression in primary care, although their coefficients were different. However, five risk factors were only included in the predictD-Spain, whilst a further four were included in the predictA-Spain. We highlight the inclusion of dissatisfaction with paid work and the perception of financial strain, which were good predictors of anxiety but not depression [39]. Furthermore, the perception of many financial difficulties by men (interaction financial strain*sex) quadrupled the incidence of anxiety at 12 months, reaching 80% (see Figure 3); which might trigger a priority prevention for these cases. This may possibly be explained by the predominant role of the male in Spanish culture to maintain the family financially. Thus, given a situation of maximum hardship the male could either be affected more or have less capacity than the female to cope adequately. Concerning the antagonistic interaction sex*age (see Figure 2), a similar result was found in a study of the prevalence of anxiety in Europe [58]. The interaction age*dissatisfaction at work also had an antagonistic effect (Figure 4), a possible reason for which is that younger persons have less work experience and may therefore have less efficient coping strategies for work problems. The fact that anxiety and depression share many risk factors could be explained in part by their frequent co-occurrence, although it is also possible that both are expressions of a latent pathological process [59]. Their different phenotypes may be associated with the interaction between different risk factors in each case, including those not measured in this study (e.g. genes, personality, coping).

The predicted probability of the onset of anxiety syndromes at 12 months in an individual could be calculated from the equation in Table 3, adding the constant and the corresponding shrunk coefficients for each patient and then inverting the “logit” of the sum obtained. As these calculations are relatively complex, the most reasonable option is a spreadsheet or a web-based calculator. Our web-based calculator is available for the onset of major depression at 12 months [39], and on the same website (http://www.rediapp.org/predict/Index.php), a calculator will be available in the coming months for the onset of anxiety syndromes at 12 months.

Our results do not address how the predictA-Spain algorithm might be implemented in primary care, though this aspect can be studied in future research. Recognition by FPs of those patients with a greater overall risk (quantitative risk information) as well as their risk factors (qualitative risk information) may lead, as in cardiovascular disease, to the development of interventions tailored for intensity (level) and specificity (profile) of risk. The FPs could inform patients about their risk and provide tailored counseling and support, thus increasing the patients' empowerment and self-efficacy perception to prevent anxiety syndromes [10].

The choice of a predicted probability cut-point for making clinical decisions depends mostly on three factors: 1) the validity of each cut-point; 2) the available evidence on the effectiveness of interventions to prevent anxiety and whether this effectiveness is greater for a given predicted probability cut-point; and 3) the available evidence on the consequences of false positives and negatives regarding patients' health and quality of life and costs for patients, health services and society. For example, if an effective intervention to prevent anxiety is implemented by FPs, since FPs are usually very busy a more specific cut-point to intervene might be preferable (e.g., a predicted probability of 13–14% or greater); although this decision should be reconsidered in light of the available data on the consequences to health and cost of false negative predictions. Nonetheless, if we provide a low cost intervention to prevent depression (e.g., an internet-based guided self-help) and the consequences linked to the false positives are acceptable, a more sensitive cut-point might be interesting (e.g., a predicted probability of 10%). Interventions to prevent anxiety disorders have been developed mainly in children and adolescents [60], [61], and less frequently in the elderly [62], [63]. However, further evaluations are needed of the effectiveness of interventions to prevent anxiety disorders in general adult populations.

## Conclusion

The predictA-Spain risk algorithm is valid to predict anxiety syndromes at 12 months. Although external validation is required, the predictA-Spain is available for use as a predictive tool in the prevention of anxiety syndromes in primary care.
